# Supplementary figures and images for: Interleukin-17 is disease promoting in early stages and protective in late stages of experimental periodontitis
Source: PLoS One. 2022 Mar 17;17(3):e0265486. doi: 10.1371/journal.pone.0265486 (PMC8929577; doi:10.1371/journal.pone.0265486)

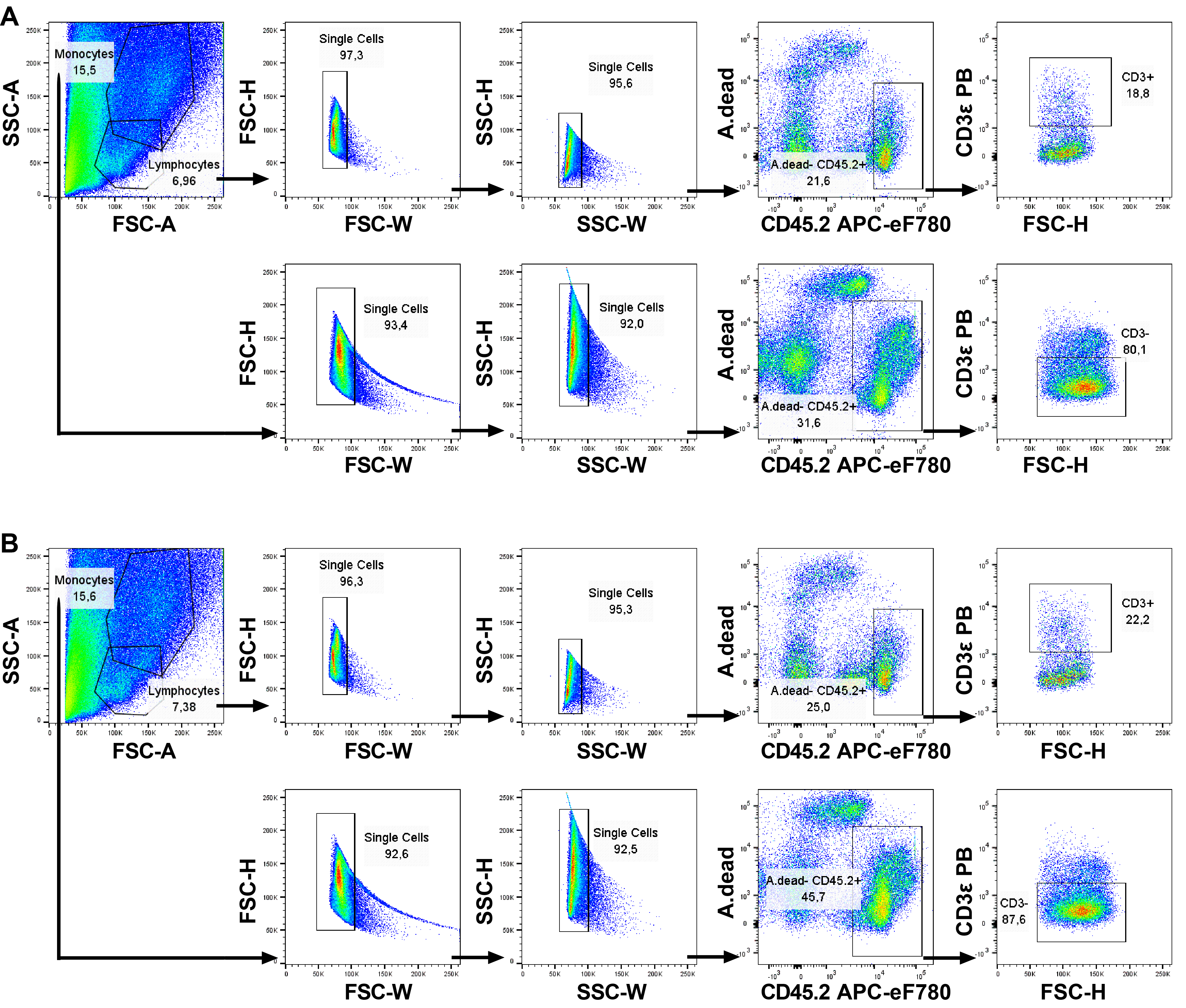

Supplement: S1 Fig — Representative gating strategy during the flow-cytometric analysis of (A) control and (B) ligature+P. gingivalis treated Tcrd-H2BeGFP mice for lymphocytes (upper panel) and neutrophils/macrophages (bottom panel). (TIF) [file pone.0265486.s001.tif]
