# Supplementary material for: Interleukin-17 is disease promoting in early stages and protective in late stages of experimental periodontitis
Source: PLoS One. 2022 Mar 17;17(3):e0265486. doi: 10.1371/journal.pone.0265486 (PMC8929577; doi:10.1371/journal.pone.0265486)
Supplement: Attachment — Submitted filename: Response to reviewers.pdf [file pone.0265486.s003.pdf]

Rebuttal letter

PONE-D-21-18861

Interleukin-17 is disease promoting in early stages and protective in late stages of experimental periodontitis

Dear Dr. Dhingra, dear reviewers,

Thank you for your careful consideration of our manuscript PONE-D-21-18861. We have the pleasure to submit a revised manuscript and to answer the questions raised by you and the reviewers.

Reply to editorial comments:
You asked to revise the manuscript regarding the PLOS one´s style requirements and file naming. We reassessed the journal-requirements and adjusted the manuscript accordingly. Since you requested to include the method of euthanasia in the methods part, we mentioned it in line 501 to 502 and as you wished, we completed the ARRIVE 2.0 Guidelines checklist. To further adhere the journal requirements, we removed the funding statement entirely from the manuscript itself. We do not want to make any changes regarding the funding statement given in the online submission form. Apart from this, we employed the Preflight Analysis and Conversion Engine (PACE) to revise the figures as you proposed.

Reply to Reviewer #1: We revised the manuscript regarding the length of the introduction and the discussion, as you suggested. We hope that the structure of the revised manuscript now meets your expectations.

Reply to Reviewer #2: We fully agree with the reviewer that this last sentence of the discussion: “in the end IL-17 has a protective function for the organism, but a damaging for the bone” is very general. According to the reviewer’s suggestion, we thus removed it from the manuscript.

Kind regards
Christoph Binz Christoph Binz

(on behalf of all authors of PONE-D-21-18861)
