# Supplementary material for: Interleukin-17 is disease promoting in early stages and protective in late stages of experimental periodontitis
Source: PLoS One. 2022 Mar 17;17(3):e0265486. doi: 10.1371/journal.pone.0265486 (PMC8929577; doi:10.1371/journal.pone.0265486)
Supplement: Attachment — Submitted filename: Response to Reviewers.pdf [file pone.0265486.s004.pdf]

Rebuttal letter

PONE-D-21-18861R1

Interleukin-17 is disease promoting in early stages and protective in late stages of experimental periodontitis

Dear Dr. Dhingra, dear reviewers,

Thank you for your careful consideration of our manuscript PONE-D-21-18861R1 and the offer to submit a revised manuscript and to answer the questions raised by the reviewers.

Reply to Reviewer #3: Thank you for making us aware of the mis-relation between the description of results versus other contents in the abstract. We reorganized large parts of the abstract and hope that it is now suitable for publication in PLOS ONE. Since you suggested a more intense and general description/discussion of IL-17 and its functions, we now added such sections in the introduction and discussion describing general effects of IL-17 as well as the role of IL-17 in several other diseases and models and giving a short comparison between the situation in mice and humans.
All changes in the revised manuscript are highlighted in yellow to facilitate peer review.

Kind regards
Christoph Binz

(on behalf of all authors of PONE-D-21-18861R1)
